# Density Functional Theory (DFT) Study of Coumarin-based Dyes Adsorbed on TiO_2_ Nanoclusters—Applications to Dye-Sensitized Solar Cells

**DOI:** 10.3390/ma6062372

**Published:** 2013-06-10

**Authors:** Corneliu I. Oprea, Petre Panait, Fanica Cimpoesu, Marilena Ferbinteanu, Mihai A. Gîrţu

**Affiliations:** 1 Department of Physics, Ovidius University of Constanţa, Constanţa 900527, Romania; E-Mails: cornel.oprea@univ-ovidius.ro (C.I.O.); p_panait@yahoo.com (P.P.); 2 Department of Theoretical Chemistry, Institute of Physical Chemistry, Bucharest 060021, Romania; E-Mail: cfanica@yahoo.com; 3 Department of Inorganic Chemistry, University of Bucharest, Bucharest 020462, Romania; E-Mail: marilena.cimpoesu@g.unibuc.ro

**Keywords:** coumarin-based dyes, density functional theory, optical spectra, titanium dioxide cluster, dye-sensitized solar cells

## Abstract

Coumarin-based dyes have been successfully used in dye-sensitized solar cells, leading to photovoltaic conversion efficiencies of up to about 8%. Given the need to better understand the behavior of the dye adsorbed on the TiO_2_ nanoparticle, we report results of density functional theory (DFT) and time-dependent DFT (TD-DFT) studies of several coumarin-based dyes, as well as complex systems consisting of the dye bound to a TiO_2_ cluster. We provide the electronic structure and simulated UV-Vis spectra of the dyes alone and adsorbed to the cluster and discuss the matching with the solar spectrum. We display the energy level diagrams and the electron density of the key molecular orbitals and analyze the electron transfer from the dye to the oxide. Finally, we compare our theoretical results with the experimental data available and discuss the key issues that influence the device performance.

## 1. Introduction

Dye-sensitized solar cells (DSSC) have attracted considerable interest over the last few years, as they offer the advantages of low fabrication costs, transparency and flexibility, when desired [1]. For such reasons, DSSCs may constitute a choice for affordable low power generation in urban areas and, in particular, a possibility of producing power generating windows [2,3].

The working principle of the DSSCs is based on light absorption in a dye anchored on TiO_2_ anatase nanoparticles, followed by transfer of the photoelectron from the dye to the wide bandgap semiconductor and through the transparent conducting oxide to the external load; at the counter electrode, the redox electrolyte facilitates the transport of the electron back to the dye and the regeneration of the sensitizer, through reduction of the triiodide ion at the counter electrode, followed by oxidation of the iodide ion at the dye [1].

The efficiency of the photovoltaic device depends strongly upon the dye and electrolyte used [4,5,6]. The highest efficiencies of power, in excess of 12%, have been obtained using Ru(II)-polypyridyl complexes [7], and values above 9% have been obtained also with metal-free organic dyes [8,9,10].

An efficient solar cell sensitizer should demonstrate (i) strong adsorption to the semiconductor surface through anchoring groups; (ii) intense absorption in the visible part of the spectrum; (iii) proper energy level alignment of the excited state of the dye and the conduction band edge of the semiconductor, as well as the redox level of the electrolyte and the ground state of the dye; (iv) fast charge transfer from the dye to the substrate, with low loss of photoelectrons; and (v) electrochemical and thermal stability [11,12]. In the attempt to find dyes satisfying these requirements, a large number of different molecules have been synthesized and characterized [11].

In this context, density functional theory (DFT) and time-dependent DFT (TD-DFT) methods have been applied to large molecules with reasonable accuracies of a few tenths of an eV, allowing for the description not only of the absorption spectrum of the dye, but also of the more complex system consisting of the dye and the TiO_2_ cluster [13,14,15,16]. The bonding of the dye on the nanoparticle and the alignment of the energy levels of the two subsystems have been successfully described by means of a DFT approach [13,16].

**Scheme 1:**
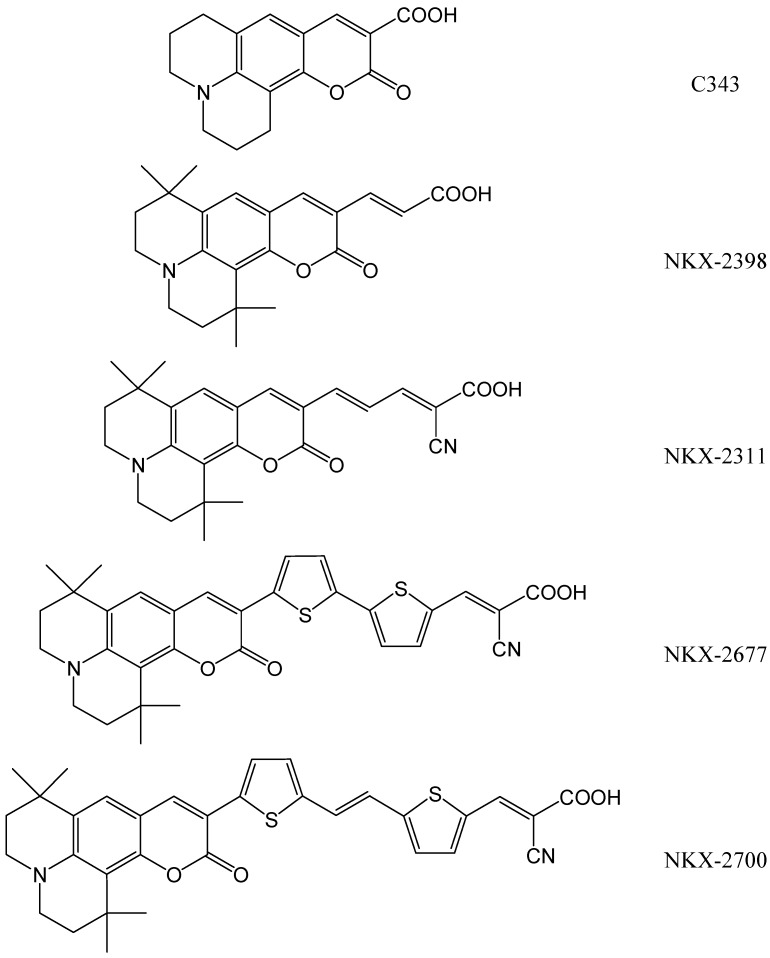
Molecular structures of some coumarin-based dyes.

We illustrate the applicability of the DFT method to study dye adsorbed on TiO_2_ clusters on several coumarin-based dyes successfully used in fabricating DSSCs. Among the metal-free organic dyes used in DSSCs, coumarin-based ones have shown good photoresponse in the visible region, long-term stability under light exposure and appropriate energy level alignment for injection into the conduction band of TiO_2_ [17]. The first promising report regarded the NKX-2311 dye, which lead to devices with a photovoltaic conversion efficiency of 5.6% [18]. Later on, the NKX-2677 dye has been used successfully as a photosensitizer in DSSCs with an even larger efficiency value of up to 7.4% [19]. The stability under sun soaking [20] and the efficiency were further improved, reaching 8.2% with NKX-2700 [21], by means of a thiophene-based bridge between the donor/acceptor parts of the push-pull structure [11]. Various other coumarin-based dyes have been synthesized and studied as TiO_2_ sensitizers, a few reviews being provided, for instance, in [11,22]. The molecular structures of coumarin-based dyes are shown in Scheme 1.

Several computational studies of coumarin-based dyes have been published, explaining some of the outstanding properties of these dyes. One of the first such study was reported by Hara *et al.* [23] and was focused on the calculation of the oxidation and reduction potentials of various early coumarin-based dyes. Performing DFT and TD-DFT calculations, Kurashige *et al.* [24] investigated the excited states of the coumarin dyes, Preat and coworkers [25] studied the electronic spectra, whereas Zhang *et al*. [26] suggested structural changes, which would improve the light absorption and energy level alignment. More recently, Sanchez-de-Armas *et al.* [27] addressed some requirements for the DSSC sensitizers, such as the position and width of the first band in the electronic absorption spectra, the absorption threshold and the LUMO energy with respect to the conduction band edge of the semiconductor. The authors also consider a small oxide cluster of (TiO_2_)_9_ and the binding of the dye to it. A study of a coumarin-based dye adsorbed on the TiO_2_ substrate was reported by Kondov *et al.* [28] on a few of the smallest dyes in the family, less interesting in the DSSC applications.

Given the interest in the coumarin-based dyes and the need to better understand the behavior of the dye adsorbed on the TiO_2_ nanoparticle, we present here results of DFT and TD-DFT studies of several complex systems consisting of a coumarin-based dye bound to a TiO_2_ cluster. The three coumarin derivatives studied here are:

(i) 1-oxo-2,3,6,7-tetrahydro-1H,5H,11H-pyrano[2,3-f]pyrido[3,2,1-ij]quinoline-10-carboxylic acid (C343),
(ii) 3-(1,1,6,6-tetramethyl-10-oxo-2,3,5,6-tetrahydro-1H,4H,10H-11-oxa-3a-azabenzo[de]anthracen-9-yl)-acrylic acid (NKX-2398) and
(iii) 2-cyano-5-(1,1,6,6-tetramethyl-10-oxo-2,3,5,6-tetrahydro-1H,4H,10H-11-oxa-3a-aza-benzo[de]anthracen-9-yl)-penta-2,4-dienoic acid (NKX-2311).

In this paper, we provide the electronic structure and UV-Vis-simulated spectra of the dyes alone, as well as adsorbed on a TiO_2_ cluster of a more realistic size, Ti_24_O_50_H_4_, to discuss the matching with the solar spectrum. We display the energy level diagrams and the electron density of the key molecular orbitals for both the dye and the dye-nanocluster system to analyze the electron transfer from the dye to the oxide. Finally, we compare our theoretical results with the experimental data available and explain the device performance of the DSSCs using these coumarin-based dyes. We also comment on the superiority of the more holistic approach, dealing with the entire dye-substrate system.

## 2. Computational Details

The structures of the dyes were optimized in both neutral and deprotonated forms by density functional theory (DFT) [29,30,31] using the generalized gradient approximation (GGA) BLYP exchange-correlation functional [32,33] and effective core potentials (ECP) for Ti atoms and double-ζ quality basis functions for all atoms via LANL2DZ [34]. For the electronic structure, single-point calculations were performed using the hybrid B3LYP functional [33,35] with the same basis set. In the case of isolated dye molecules, extra polarization functions required for more accurate electronic densities were included via the DZVP [36] basis sets. Singlet-to-singlet electronic transitions were calculated by time-dependent-DFT (TD-DFT) [37], their number varying from 20 to 100 depending on the size of the system. The solvent effect was accounted for by employing the polarizable continuum model (PCM) [38,39], which treats the solvent as a homogeneous dielectric medium. The cavity used in the PCM calculation was built from spheres centered on heavy nuclei, based on the United Atom for Hartree-Fock procedure described in [39]. All calculations were performed with the GAUSSIAN03 quantum chemistry package [40].

## 3. Results and Discussion

This section is divided in six parts, dealing with the main criteria for the DSSC sensitizers. Three of these subsections refer to the dye alone, one to the TiO_2_ nanoclusters and the last two to the more complex system consisting of the dye-oxide couple.

**Figure 1:**
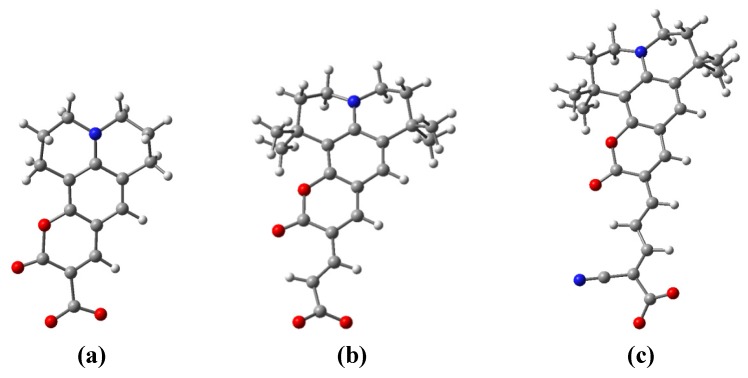
Optimized structure of the dyes (**a**) C343; (**b**) NKX-2398 and (**c**) NKX-2311 in their deprotonated form calculated at density functional theory (DFT)/BLYP/LANL2DZ level. The proton missing belongs to the anchoring carboxyl group.

The optimized geometrical structures of all the three dyes have been previously reported by other authors [23,24,26] and fall out of the focus of the present report. We only state that the structures are in agreement with the ones already presented. In order to better describe the dye bound to the oxide, we also performed calculations of the deprotonated dyes, by taking away the proton from the anchoring carboxyl group. The optimized structures are shown in Figure 1.

### 3.1. Absorption Spectrum

**Figure 2:**
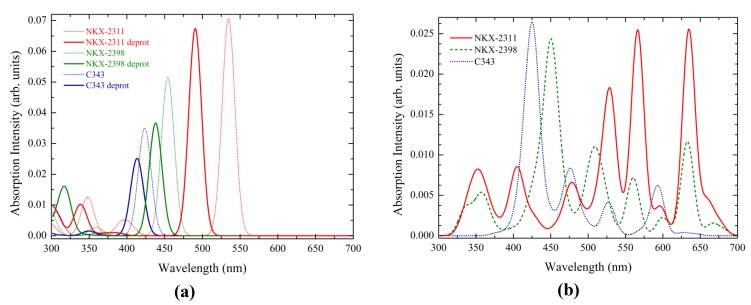
Simulated UV-Vis absorption spectra of (**a**) neutral and deprotonated dyes and of (**b**) dyes adsorbed on the substrate (b), calculated by TD-DFT in water. The spectral lines were convoluted with Gaussian distributions of 20 nm linewidth at half maximum.

One of the most important requirements for a dye, to be used in DSSCs, is to have an absorption spectrum matching the solar irradiation spectrum. Therefore, we calculated by TD-DFT the UV-Vis-simulated absorption spectra (see Figure 2) in water solvent for all three dyes in both neutral and deprotonated forms. Similar calculations were performed in solvents used in the actual DSSCs, like ethanol and methanol and the results are displayed in the Supplementary Materials. The spectra that result when using a continuum model, such as PCM, are very similar for all solvents, particularly in the visible region of the spectrum.

The spectra shown in Figure 2 indicate a systematical blue shift of the peaks of the deprotonated dyes with respect to the neutral ones. This result has been observed also for ruthenium(II)-bipyridine dyes [41], being attributed to the decrease of the static dipole moments of the excited states of deprotonated dyes, leading to a different amount of solvent stabilization in ground and excited states [42].

The spectra of the more complex dyes have absorption bands situated in the longer-wavelength region relative to the peak for C343, which is located at 424 nm for the neutral and 414 nm for the deprotonated form, below the experimental value of 442 nm [23]. Other authors obtained similar values of 401 nm [24] or 419 nm [27] for the neutral and 384 nm [24] for the deprotonated dye.

The absorption peaks for NKX-2398 are located at 454 nm for the neutral and at 438 nm for the deprotonated form, compared to the 451 nm observed experimentally [23]. Calculations by other authors lead to even lower values of 422 nm [26].

Similarly, for NKX-2311, we obtained 535 and 491 nm, for the neutral and deprotonated forms, respectively. The experimental value is 504 nm [23]. Other calculations provided comparable values, of 510 nm [24], 506 nm [26] and 528 nm [27] for the neutral and 469 nm [24] for the deprotonated form.

We note at this point that all the bands mentioned above are due to transitions between the highest occupied and the lowest unoccupied molecular orbitals (HOMO → LUMO). As we shall see in Section 3.3, all these transitions have a π → π* nature.

We also note that, compared to C343, the extra methane unit and carboxyl group on NKX-2398 cause a slight redshift, which is more pronounced for NKX-2311, with a cyanoacetic acid unit connected directly to the conjugated chain. Connecting the cyanoacetic acid moiety, to the coumarin framework, via a conjugated chain, leads to a considerable red shift in the absorption properties of the dyes, a shift that is desirable for harvesting light from the solar spectrum [23].

The simulated optical spectra of the dye adsorbed onto the TiO_2_ nanocluster show more complex features, displaying strong absorption even at higher wavelengths. The main peaks of C343 and NKX-2398 remain in the blue region, whereas for NKX-2311, the weight of the spectrum changes with stronger and more evenly spread absorption in the green and red regions of the spectrum. We will return to clarify the nature of the peaks in Section 3.6. Here, we just make two notes. First, we anticipate that the new bands at lower energy/higher wavelengths occur because some transitions that initially were forbidden now become allowed, due to the mixed character of the orbitals located in the gap, which also have a contribution from the TiO_2_ substrate. Second, consistent with the UV-Vis spectra of the dyes in solution, the adsorbed dyes can be ordered in terms of the matching with the solar irradiation spectrum [43] as follows: NKX-2311 > NKX-2398 > C343.

### 3.2. Energy Level Alignment and Electron Transfer

**Figure 3:**
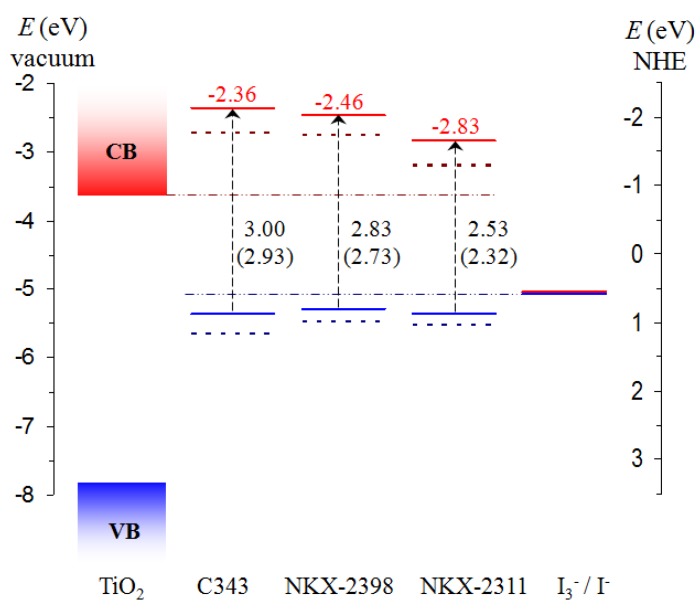
Energy diagram showing the ground state (blue) and the excited state (red) energies calculated in solution by DFT and time-dependent (TD)-DFT (B3LYP/LANL2-DZVP) methods for the deprotonated (solid line) and neutral (dotted line) dyes. The conduction (red) and valence (blue) bands of the TiO_2_, as well as the redox level of the I_3_^−^/I^−^ electrolyte [16], are also shown on the left and right hand side, respectively. The energy of the lowest singlet-to-singlet transition for the deprotonated (neutral) dyes are stated next to the transition lines, whereas the energy of the excited state is displayed above the corresponding levels.

To investigate the possibility of the charge injection into the semiconductor and of the regeneration of the dye, we performed DFT calculations providing the energy level alignment of the dye with the substrate and the electrolyte. The energy of the ground and the excited states of the dyes, together with the energies of the valence (VB) and conduction (CB) band edge for TiO_2_ (see Section 3.4, below), and the redox level of the I_3_^−^/I^−^ electrolyte [1] are displayed in Figure 3. The dotted lines are drawn as aids to the eye for an easier examination of the energy level alignment. The energy of the excited state is obtained based on the sum between the energy of the ground state (HOMO) and the energy of the lowest singlet-to-singlet transition [16]. Next to the transition arrow, we display the singlet-to-singlet transitions between the deprotonated (neutral) dyes. The values on the axes in Figure 3 are relative to the vacuum (on the left axis) and to the normal hydrogen electrode (NHE, on the right axis) [1,44].

For all dyes, the LUMO lies above the conduction band edge of TiO_2_, making possible the transfer of the photoelectron from the dye to the semiconducting oxide. In addition, the HOMO of all dyes lies below the redox level of the electrolyte, allowing for the transfer of the electron to the dye and its regeneration.

The driving force of the electron injection from the dye into the semiconductor is the energy difference between the excited state of the dye and the conduction band edge of the oxide [45]. In the cases studied here, the energy differences for the deprotonated (neutral) dyes are 1.26 eV (0.81 eV) for C343, 1.17 eV (0.88 eV) for NKX-2398 and 0.79 eV (0.43 eV) for NKX-2311. Other authors obtained relatively similar values, 1.24 eV for NKX-2398 and 0.67 eV for NKX-2311 nm [26] or 1.033 eV for C343 and 0.414 eV for NKX-2311 [27].

We note that it is stated (based on a theoretical result obtained under some simplifying approximations) that the injection rate increases when the adsorbate excited state is further above the conduction band edge [46]. A consequence of this statement would be that a larger driving force would be desirable for a more rapid electron injection rate and a higher overall device efficiency [26]. Although, under larger driving forces, the speed of the electron injection into the semiconductor may increase [47], such increases are not always reflected in higher efficiencies of the device and not even in larger short circuit current densities. For instance, in the case of the dyes studied here, both the photovoltaic conversion efficiency, *η*, and, more importantly, the short circuit current density, *J*_sc_, of the devices are negatively correlated with the driving force. The experimental values for C343, NKX-2398 and NKX-2311 are 4.1, 11.1 and 15.2 mA/cm^2^ for *J*_sc_ and 0.9%, 3.4% and 5.2% for *η* [23], in clear contradiction with the decreasing values of the driving force [23,26,27].

The apparent contradiction between theoretical expectations and experimental results is not surprising for the device efficiency, which depends on a wider variety of requirements, as already mentioned in the introduction [11]. Even the short circuit current density is influenced not just by the energy difference between the excited state of the dye and the conduction band edge, but by other factors as well, such as the light harvesting efficiency (correlated with the absorption coefficient) and the propensity for electron transfer, not to mention recombination and leakage currents in the device.

An inverse relation between the driving force for the electron injection and the short-circuit current density has been observed in other cases, as well. An example is provided by tetrahydroquinoline-based dyes [48]; another is offered by Mordant Yellow-10, for which the devices displayed decreasing *J*_sc_ with increasing distance of the excited state from the conduction band edge [49,50].

The driving force of the electron transfer cannot be increased indefinitely without jeopardizing other factors that influence the performance of the photovoltaic cell. Raising the excited state level of the dye with respect to the conduction band edge of the oxide tends to also raise the ground state level, in order to keep the light harvesting properties. In turn, the lifting of the ground state of the dye is restrained by the condition that the electrolyte redox level remains higher, in order to allow for the regeneration of the dye. Consequently, statements that a larger electron injection driving force is desirable for a higher overall device efficiency [26] should be carefully scrutinized.

### 3.3. Electron Transfer

**Figure 4:**
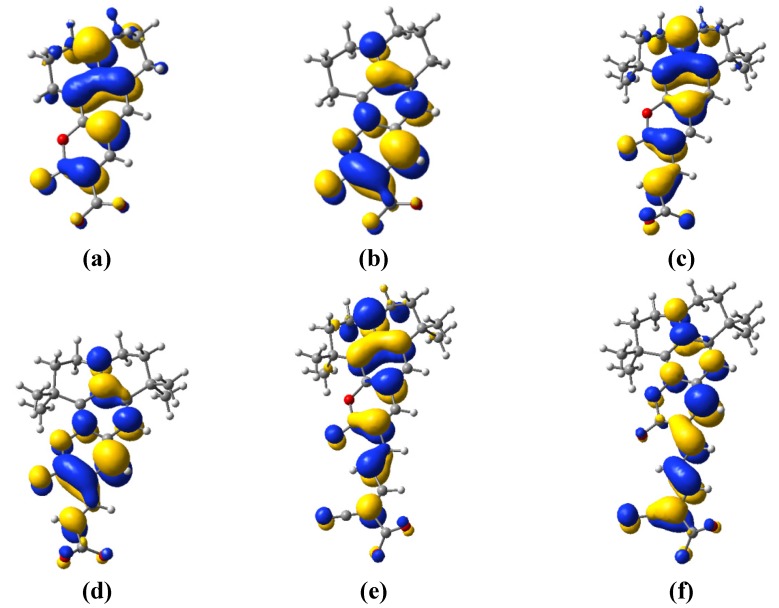
Isodensity surfaces (0.03 e/bohr^3^) of the key molecular orbitals of C343, (**a**) HOMO; (**b**) LUMO, NKX-2398; (**c**) HOMO; (**d**) LUMO and NKX-2311; (**e**) HOMO; (**f**) LUMO in deprotonated form calculated at the DFT/B3LYP/DZVP level in water.

The energy level alignment shows that both the electron injection into the conduction band of the oxide and the regeneration of the sensitizer are possible. However, to further explore the likelihood of these processes, it is useful to also look at the electron densities of the highest occupied (HO) and lowest unoccupied (LU) molecular orbitals (MO). As shown in Figure 4, for all dyes and all molecular orbitals, a considerable electron density is found in the coumarin part of the molecule. Moreover, it can be seen that all the HOMOs have π character and all the LUMOs π*, justifying the claim in Section 3.1 that the main absorption band is due to a π → π* transition.

To further analyze the propensity for electron transfer of the three dyes, we determined the contribution to the electron density of each atom and added these weights for the donor and acceptor groups. We considered that the donor unit consists of the coumarin-quinolizine groups, whereas the acceptor units consist of the carboxyl group and, where appropriate, the π conjugated bridge. The values are reported in Table 1.

As expected, due to the absence of the π-conjugated bridge, in the case of the C343 dye, most of the electron density is delocalized over the donor group, particularly on the coumarin. The light absorption pushes the electron density towards the acceptor unit only slightly. In the case of NKX-2398, in the excited state, the electron density is kept away from the acceptor, suggesting that the electron injection to the oxide is less favorable under these conditions. In contrast, for NKX-2311, the electron density on the acceptor unit more than doubles in the excited state compared to the ground state. The presence of the π-conjugated bridge and of the cyanoacetic acid moiety encourages the electron transfer.

**Table 1:** Contributions of the donor and acceptor groups to the electron density of the main molecular orbitals, calculated at the DFT/B3LYP/DZVP level.

Dye	MO	Contribution to electron density of the donor unit (%)	Contribution to electron density of the acceptor unit (%)	Total (%)
C343	HOMO	98.473	1.527	100
	LUMO	97.572	2.428	100
NKX-2398	HOMO	85.845	14.155	100
	LUMO	90.806	9.194	100
NKX-2311	HOMO	79.586	20.414	100
	LUMO	57.181	42.819	100

### 3.4. Titania Nanoclusters

So far, we have studied the properties of the free dye, independent of the oxide to which it binds. Insight into the dye adsorption to the substrate, the absorption spectrum and its matching to the solar spectrum, as well as into the electron transfer, can be more readily obtained studying a more complex system, consisting of the dye bound to the TiO_2_ cluster. In this context, DFT methods have proven to provide a reasonable compromise between accuracy and the ability to treat large systems [13,16].

**Figure 5:**
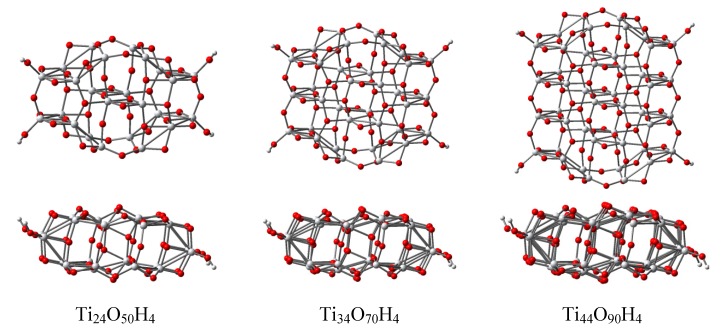
Top and lateral views of the optimized structures of the Ti_24_O_50_H_4_, Ti_34_O_70_H_4_ and Ti_44_O_90_H_4_ nanoclusters modeling the anatase titania (101) surface. The geometry optimization was performed at the DFT/BLYP/LANL2DZ level.

We modeled TiO_2_ anatase nanoparticles by means of geometry optimized clusters with molecular formulae Ti_24_O_50_H_4_, Ti_34_O_70_H_4_ and Ti_44_O_90_H_4_ (see Figure 5). The starting geometry for the structural optimization of the clusters originated in the experimental structure of the {101} anatase titania surface, which is the most common [51,52]. We noticed that calculations of finite models of different sizes with the geometry resulted from X-ray diffraction experiments [53] lead to a narrow spacing of about 1.3 eV between occupied and unoccupied states, caused by the presence of surface states in the band gap of the semiconductor. The surface tensile stress was released for TiO_2_ slabs by the insertion of TiO_3_ species, followed by geometry optimization [54].

To avoid the problem of the surface states in the gap, we performed geometry optimization of model clusters with a slight deviation from the TiO_2_ stoichiometry, introducing four hydrogen atoms to terminate the dangling bonds at the periphery. This approach resulted in compact structures with 4-, 5- and 6-fold coordinated Ti ions, together with two- and three-fold coordinated oxygen atoms. For each model, the four protons ensure for each of the neutral finite models the removal of dangling oxygen states from the band gap.

Following the geometry optimization, the structure is slightly distorted, to minimize the surface stresses. The typical distances and cluster sizes for the optimized and the experimental (bulk) [51] structures are displayed in Table 2. The average value of the Ti–O distance has a small decrease for all clusters, but the distribution of these distances widens significantly compared to the bulk. On average, the deformation of the structure changes angles and distances, such that the larger distances tend to be compensated by smaller ones. The length and the width of the clusters have inverse variations, the former increasing, while the latter decreases. The relative variation of the cluster distances compared to the values in the bulk is less than 6%, typically around 4%.

**Table 2:** Average Ti–O distance and its standard deviation, as well as the cluster length and width, in Å, of the DFT/BLYP/LANL2DZ optimized structures compared to the experimental value of the bulk TiO_2_ [51].

Parameter	Ti_24_O_50_H_4_	Ti_34_O_70_H_4_	Ti_44_O_90_H_4_	TiO_2_ (bulk)
*r*(Ti–O)	1.909	1.908	1.916	1.950
*σ_r_*_(Ti–O)_	0.133	0.101	0.105	0.022
Length	12.76	12.54	12.50	12.04
Width	7.39	10.91	14.34	–
Width (bulk)	7.59	11.37	15.14	–

The geometry relaxation lead to band gaps of 4.24 to 4.55 eV, significantly larger than the experimental value of ~3.2 eV for anatase titania [1]. A quantity that crucially influences the performance of DSSCs is the position of the conduction band edge, which can be determined by DFT calculations, *E*_CB_ or based on the energy of the electronic transition, as an oxidation potential [16], CBOP. The actual value depends on the cluster size and on the basis set used, as it can be seen in Table 3.

**Table 3:** Conduction band edge, in eV, calculated for clusters of various sizes in water, using DFT and TD-DFT, the B3LYP functional and different basis sets.

Basis set	Ti_24_O_50_H_4_		Ti_34_O_70_H_4_		Ti_44_O_90_H_4_		(TiO_2_)_38_ [16]	
	*E*_CB_	CBOP	*E*_CB_	CBOP	*E*_CB_	CBOP	*E*_CB_	CBOP
3-21G*	−3.20	−3.98	−3.41	−4.12	−3.51	−4.14	−2.77	−3.35
DZVP	−3.86	−4.63	−3.98	−4.67	−4.02	−4.69	−3.44	−4.04

As the size of the cluster is an important factor in the economy of the simulation, larger sizes requiring significantly higher computer resources and longer computation times, we consider that the Ti_24_O_50_H_4_ cluster is appropriate for calculations involving coumarin dyes. Larger clusters, such as (TiO_2_)_82_, might be more appropriate when dealing with larger dye molecules, particularly the typical ruthenium(II) complexes [55].

**Figure 6:**
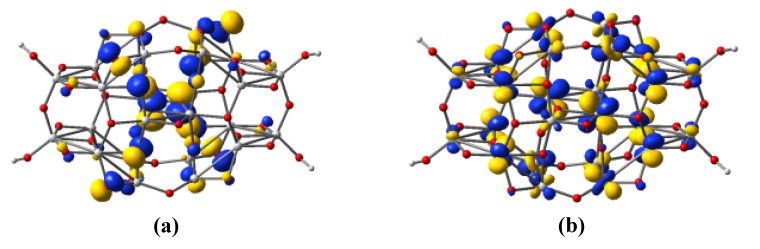
Isodensity surfaces (0.03 e/bohr^3^) of the key molecular orbitals of the Ti_24_O_50_H_4_ cluster, calculated at the DFT/B3LYP/LANL2DZ level in water: (**a**) highest occupied valence band orbital; (**b**) lowest unoccupied conduction band orbital.

The electronic structure of the Ti_24_O_50_H_4_ cluster is shown in Figure 6. The highest occupied valence band (VB) state consists mostly of oxygen 2*p* atomic orbitals, whereas the lowest unoccupied state of the conduction band is formed by titanium 3*d* atomic orbitals.

**Figure 7:**
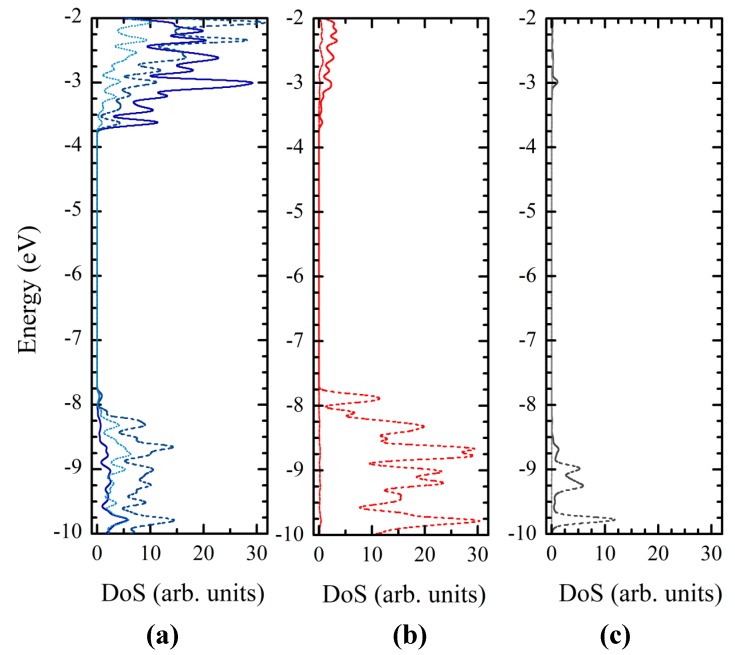
Density of states of the Ti_24_O_50_H_4_ cluster, calculated at the DFT/B3LYP/LANL2DZ level in water: (**a**) contribution of Ti atoms (a); O atoms (**b**) and –OH groups (**c**). The different lines show the contribution of the *d* orbitals (solid), *p* orbitals (dashed) and *s* orbitals (dotted).

The nature of the states in the valence and conduction band can be more easily determined from Figure 7, which displays the density of states (DOS). The valence band is dominated by the *p* atomic orbitals of O with a little contribution from the *p* atomic orbitals of Ti. The key contribution in the conduction band comes from the Ti orbitals, especially the *d* and *p* ones, the involvement of the oxygen atoms being very small, even for the *p* orbitals. Important to note is that the introduction of the hydrogen atoms that end four dangling bonds have a minor contribution to the DOS, but play an important role in removing the surface states from the gap.

### 3.5. Dye Adsorption 

The anchoring modes of the dye to the TiO_2_ surface are of crucial importance, the bonding type and the extent of electronic coupling between the dye-excited state and the semiconductor unoccupied states, directly influencing the overall cell performance [1,11,56]. Starting from the pioneering work by Vittadini *et al.* [57] on the formic acid adsorption on the TiO_2_ anatase (101) surface, a number of theoretical studies on the dye adsorption modes on the titania surface have been published [4,13,14,15,16,58,59,60]. The calculations show that for the organic dyes bearing a carboxylic acid as the anchoring group, the preferred adsorption mode is bidentate bridging, with one proton transferred to a nearby surface oxygen, while the monodentate anchoring is usually predicted to be less stable, although some dependency of the relative stability of these two anchoring modes on the employed computational methodology can be outlined [53,54].

**Figure 8:**
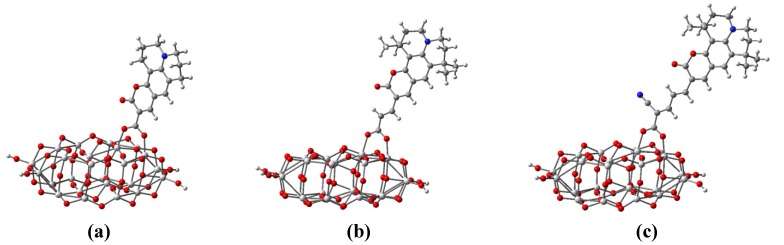
Optimized structure of the dye-oxide complex systems: (**a**) C343–Ti_24_O_50_H_4_; (**b**) NKX-2398–Ti_24_O_50_H_4_; (**c**) NKX-2311–Ti_24_O_50_H_4_, at the DFT/BLYP/LANL2DZ level.

Our calculations of the three coumarin-based dyes of interest here showed that the preferred adsorption mode is indeed the bidentate bridging. Starting the optimization from the other types of binding (monodentate ester-like or bidentate chelating), finally lead to the same bidentate bridging configuration (see Figure 8).

The relevant distances and angles relevant to the binding of the dye to the Ti_24_O_50_H_4 _cluster are presented in Table 4. The two Ti–O bond lengths are different by about 7% for all three dyes. The shorter distances were found for NKX-2398, suggesting a strong binding.

**Table 4:** Distances, in Å, and angles, in degrees, relevant to the binding of the dye to the cluster, after geometry optimization at the DFT/BLYP/LANL2DZ level.

Parameter	C343-Ti_24_O_50_H_4_	NKX-2398-Ti_24_O_50_H_4_	NKX-2311-Ti_24_O_50_H_4_
*r*(Ti–O1)	1.977	1.969	1.986
*r*(Ti–O2)	2.128	2.115	2.138
*θ*	105.2	113.5	122.2
*τ*	16.5	23.5	32.6

The angle, *θ*, was chosen between the direction uniting the C atom of the carboxyl group and the N atom of the quinolizine group and the direction of the two binding Ti atoms. It shows the inclination of the dye with respect to the cluster. We note that *θ* increases significantly from C343 to NKX-2398 and NKX-2311, indicating the higher tilting of the dye. The angle, *θ*, is sensitive to the difference in the two binding distances.

The tilting of the plane of the dye with respect to the cluster surface is measured by angle, *τ*. More specifically, angle *τ* is the angle between the already mentioned direction connecting the C atom of the carboxyl group and the N atom of the quinolizine unit and the normal to the plane of the three adjacent five-connected Ti atoms (two of which are involved in the binding). Angle, *τ*, influences the overlap between the π or π* orbitals of the dye and the *d*_z2_ orbital of the titanium ion (a larger tilting of the molecular plane leads to higher overlap), which, in turn, can affect the electron transfer. Noting that angle, *τ*, increases from C343 to NKX-2398 and NKX-2311, we have now reason to believe that the electron injection into the semiconductor is facilitated in NKX-2311.

At the end of this section, we note that based on a combined experimental and theoretical study of a family of organic dyes, we reported elsewhere a discussion of the relative importance of the dye adsorption criterion with respect to the spectral matching and energy level alignment [61].

### 3.6. Optical Properties of Adsorbed Dyes

**Figure 9:**
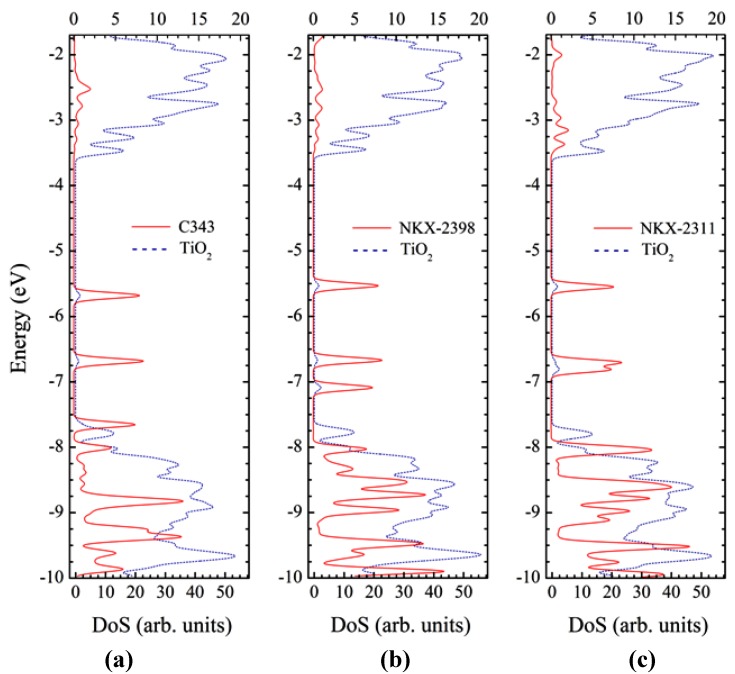
Density of states of the dye-Ti_24_O_50_H_4_ cluster complex systems, calculated at the DFT/B3LYP/LANL2DZ level in water: (**a**) C343–Ti_24_O_50_H_4_; (**b**) NKX-2398–Ti_24_O_50_H_4_; (**c**) NKX-2311–Ti_24_O_50_H_4_. Bottom and top scales are for the nanocluster and dye contributions, respectively.

**Figure 10:**
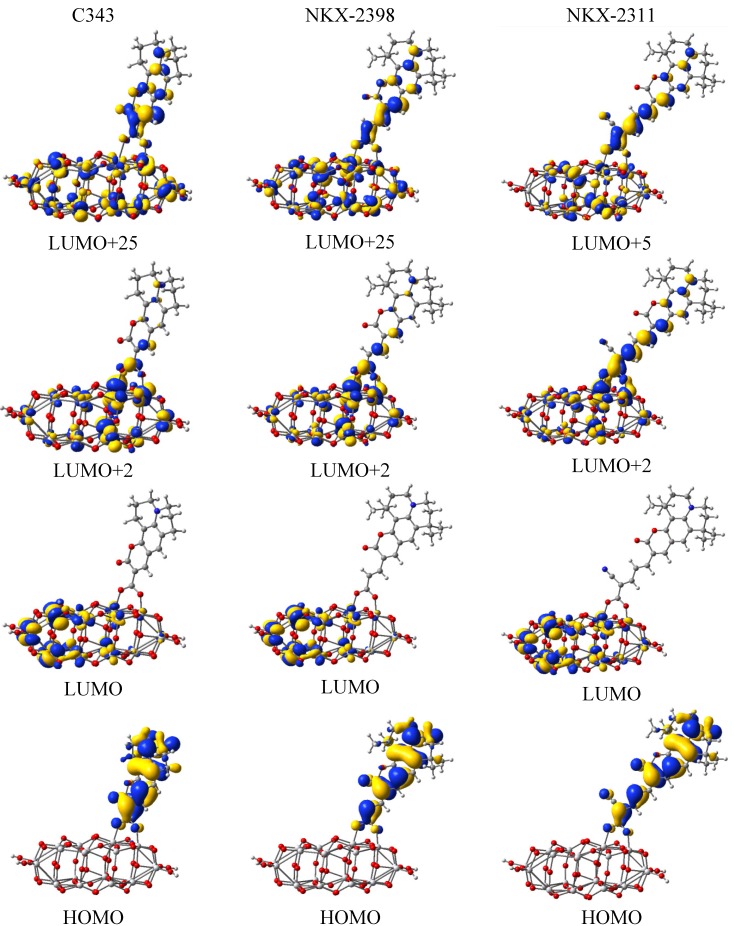
Isodensity surfaces (0.02 e/bohr^3^) of the key molecular orbitals of the dyes adsorbed on the TiO_2_ nanocluster calculated at the DFT/B3LYP/LANL2DZ level in water.

The density of states for each of the three dyes is represented in Figure 9. For all three systems, the valence band has a mixed character with significant contributions from both the dye and the cluster. In contrast, the conduction band has dominating contributions from the titania cluster. In order to better understand the nature of the orbitals, we represented in Figure 10 the electron density of the most important states.

One of the key orbitals from the perspective of DSSCs is the HOMO of the entire system, which, for all three dyes, is situated in the gap, significantly higher than the valence band edge located at about −7.8 eV. As it can be seen from Figure 10, in each case, the HOMOs are strongly localized on the dye, corresponding to the ground state of the free dye. They are located at −5.68 eV for C343, −5.53 eV for NKX-2398 and −5.54 eV for NKX-2311.

Moving higher on the energy scale, the next key state is the LUMO of the entire system, which, for all three dyes, is strongly localized on the cluster (see Figure 10), corresponding to the conduction band edge of the oxide. The LUMOs are located at −3.47 eV for C343, −3.45 eV for NKX-2398 and −3.47 eV for NKX-2311.

The dye contribution to the DOS in the conduction band of all three systems is revealed by some weak peaks. One set of such peaks corresponds to the LUMO+2 state, which has mixed character, with electron density delocalized over both the dye and the oxide. The energy of these states is −3.29 eV for C343, −3.29 eV for NKX-2398 and −3.37 eV for NKX-2311.

To better understand the optical spectra in Figure 2, we need to also look at the few states situated in the gap below the HOMO, states that, as expected, are well localized on the dye. In the case of C343, HOMO-1 is at −6.68 eV, whereas HOMO-2 is near the band edge of the valence band, at −7.65 eV. Compared to C343, for NKX-2398 HOMO-1 is at about the same energy, at −6.67 eV, whereas HOMO-2 lies higher, at −7.09 eV. The tendency is continued with NKX-2311, for which HOMO-1 is situated at −6.70 eV and HOMO-2 much closer, at −6.80 eV.

The local maxima in the dye DOS correspond to states with π* character and with considerable electron density distributed over the dye. These states are LUMO+25, LUMO+23 and LUMO+5, their energy being −2.52 eV, −2.53 eV and −3.16 eV for C343, NKX-2398 and NKX-2311, respectively.

Now, we can return to the simulated optical spectra of the dye adsorbed onto the substrate, displayed in Figure 2, and discuss the nature of the absorption bands by analyzing the contributions of the states involved in transitions. We note that important similarities can be observed for all dyes. In all spectra, there are four major bands shifted to higher wavelengths when we compare C343 to NKX-2398 and NKX-2311.

The first (low energy/high wavelength) bands (635 nm for NKX-2311, 633 nm for NKX-2398 and 593 for C343) correspond for all three systems to HOMO → LUMO+2 transitions. The intensity is strongest for NKX-2311, whose LUMO+2 has the largest electron density localized on the dye atoms. These first bands did not appear in the spectra of the free dyes, as the LUMO+2 orbitals originate from the cluster and now have mixed character, with some contribution from the dyes (see Figure 10). It is worth noting that these first bands also show a shoulder, from HOMO → LUMO excitations (charge transfers from the dye to the cluster), which were also missing from the spectra of the dyes. These last excitations are responsible for the widening of the first band of the spectra in the red region.

The similarities between the three systems stop below the first bands. For NKX-2311, the next two bands, at 566 nm and 529 nm, are due to HOMO → LUMO+5 and HOMO → LUMO+9 transitions, respectively, both to orbitals with mixed character, with some contribution from the dye. In contrast, the next two bands for the other dyes originate from transitions to states that are strongly localized on the cluster. For NKX-2398 and C343, the transitions at 560 nm and 527 nm, respectively, are HOMO → LUMO+6, whereas those at 509 nm and 476 nm are HOMO → LUMO+13.

The last band in the visible region is located at 487 nm for NKX-2311. It represents a HOMO → LUMO+19 transition, the final state having some electron density over the carboxyl group. The other two dyes have transitions at 450 nm and 425 nm, both HOMO → LUMO+25 excitations, towards states with considerable π* dye contribution.

At the end of this section, it is worth noting that the more holistic approach that allows the simulation of the dye and cluster together has, among others, the key advantage of explaining the lower energy transitions in the optical spectra. Allowing for the mixing of oxide and dye states this approach goes significantly beyond the separate treatment.

## 4. Conclusions 

In this work, we have applied a computational approach, which combines DFT and TDDFT techniques to study how three typical coumarin-based dyes fulfil the criteria for TiO_2_ sensitizing dyes for DSSCs. We examined the absorption spectra, which influence the light harvesting properties of the dyes, the energy level alignment between the dye, the oxide and the electrolyte, which affect the electron injection and the dye regeneration and the adsorption of the dye to the substrate, determining the charge transfer.

Based on our TD-DFT calculations, we discussed the absorption spectrum of the isolated neutral and deprotonated dyes. The simulated UV-Vis absorption peaks of all coumarin dyes are in good agreement with the experimental data. Due to the better matching to the solar irradiance spectrum, we concluded that NKX-2311 has superior light harvesting properties to both NKX-2398 and (especially to) C343.

The analysis of the energy level alignment showed that for all free dyes, the LUMO lies above the conduction band edge of TiO_2_, making possible the electron injection into the semiconducting oxide. In addition, the HOMO of all dyes lies well below the redox level of the electrolyte, allowing for the transfer of the electron to the dye and its regeneration.

We commented on the inverse relation between the short-circuit current density and the energy difference between the excited state of the dye and the conduction band edge of the oxide, which is considered the driving force for the electron transfer. Based on the experimental values available, we showed that, in the case of the dyes studied here, the short circuit current densities, *J*_sc_, of the devices are negatively correlated with the driving force, as the short circuit current density is influenced not just by the energy difference between the excited state of the dye and the conduction band edge, but by other factors as well, such as the light harvesting efficiency and the propensity for electron transfer, not to mention recombination and leakage currents in the device. Moreover, we pointed out that the driving force of the electron transfer cannot be increased indefinitely without jeopardizing other factors that influence the performance of the photovoltaic cell. Raising the excited state level of the dye with respect to the conduction band edge of the oxide tends to also raise the ground state level, to preserve the light harvesting properties. However, the ground state of the dye is restrained by the condition that the electrolyte redox level remains higher, to allow for the regeneration of the dye.

We discussed the charge transfer of the photoelectron from the excited state of the dye to the semiconductor based on an analysis of the electron density distribution over the ground and excited states of the dye. The transfer is hindered for NKX-2398, but facilitated for NKX-2311, due to the tendency of the electron density to be localized closer to the COO– group (and the substrate) upon excitation by light absorption.

We modeled the TiO_2_ nanoparticles with clusters, which we used to further study the binding of the dye to the oxide. The optimized geometry allowed structure-property correlations, since some particular distances and angles can influence the orbital overlap and, consequently, the charge transfer. The TD-DFT calculation of the UV-Vis simulated spectra of the dye-oxide system allowed the assignment of the transitions and a more rigorous comparison of the three dyes, demonstrating the superiority of the NKX-2311 dye. The DFT calculation of the density of states clarified the contributions of the dye and of the oxide, allowing the discrimination of the contributions of various types of atomic orbitals (*s*, *p* or *d*).

When combining the criteria discussed, it followed that C343 would be the dye with the lowest efficiency, because of its poor matching with the solar spectrum. Furthermore, since both NKX-2398 and NKX-2311 verify the energy alignment requirements and the former has poorer optical absorption and electron transfer properties, it remains that the latter is expected to be a higher performer as a DSSC sensitizing dye.

Finally, the comparison of the results obtained for the free (non-interacting) dye with those obtained for the combined dye-oxide system showed that in simple cases, the former provides very useful first order results, reasonably close to those of the later. The superiority of the more holistic approach comes from the binding information it provides, as well as from its ability to deal with states that mix dye and oxide character. In any case, the agreement of our results with available experimental data indicates that the present approach has explanatory/predictive power and can be an efficient tool to help in the optimization of dye-sensitized solar cells.

## Supplementary Files

Supplementary File 1

## References

[1] Gratzel M. (2001). Photoelectrochemical cells. Nature.

[2] Ahn K.S., Yoo S.J., Kang M.S., Lee J.W., Sung Y.E. (2007). Tandem dye-sensitized solar cell-powered electrochromic devices for the photovoltaic-powered smart window. J. Power Sources.

[3] Baetens R., Jelle B.P., Gustavsen A. (2010). Properties, requirements and possibilities of smart windows for dynamic daylight and solar energy control in buildings: A state-of-the-art review. Sol. Energy Mater. Sol. Cells.

[4] Nazeeruddin M.K., de Angelis F., Fantacci S., Selloni A., Viscardi G., Liska P., Ito S., Bessho M., Gratzel T. (2005). Combined experimental and DFT-TDDFT computational study of photoelectrochemical cell ruthenium sensitizers. J. Am. Chem. Soc..

[5] Peter L.M. (2011). The gratzel cell: Where next?. J. Phys. Chem. Lett..

[6] Hardin B.E., Snaith H.J., McGehee M.D. (2012). The renaissance of dye-sensitized solar cells. Nat. Photon..

[7] Yella A., Lee H.W., Tsao H.N., Yi C., Chandiran A.K., Nazeeruddin M.K., Diau E.W.G., Yeh C.Y., Zakeeruddin S.M., Gratzel M. (2011). Porphyrin-sensitized solar cells with cobalt (II/III)-based redox electrolyte exceed 12 percent efficiency. Science.

[8] Ito S., Zakeeruddin S.M., Humphry-Baker R., Liska P., Charvet R., Comte P., Nazeeruddin M.K., Pechy P., Takata M., Miura H. (2006). High-efficiency organic-dye-sensitized solar cells controlled by nanocrystalline-TiO_2_ electrode thickness. Adv. Mater..

[9] Hwang S., Lee J.H., Park C., Lee H., Kim C., Park C., Lee M.H., Lee W., Park J., Kim K., Park C., Kim N.G. (2007). A highly efficient organic sensitizer for dye-sensitized solar cells. Chem. Commun..

[10] Mishra A., Fischer M.K.R., Bauerle P. (2009). Metal-free organic dyes for dye-sensitized solar cells: From structure: Property relationships to design rules. Angew. Chem. Int. Ed..

[11] Hagfeldt A., Boschloo G., Sun L., Kloo L., Pettersson H. (2010). Dye-sensitized solar cells. Chem. Rev..

[12] Hamann T.W., Jensen R.A., Martinson A.B.F., van Ryswyk H., Hupp J.T. (2008). Advancing beyond current generation dye-sensitized solar cells. Energy Environ. Sci..

[13] De Angelis F., Fantacci S., Selloni A., Grätzel M., Nazeeruddin M.K. (2007). Influence of the sensitizer adsorption mode on the open-circuit potential of dye-sensitized solar cells. Nano Lett..

[14] Hagberg D.P., Yum J.H., Lee H.J., de Angelis F., Marinado T., Martin Karlsson K., Humphry-Baker R., Sun L., Hagfeldt A., Gratzel M. (2008). Molecular engineering of organic sensitized for dye-sensitized solar cells applications. J. Am. Chem. Soc..

[15] De Angelis F., Fantacci S., Sgamellotti A. (2007). An integrated computational tool for the study of the optical properties of nanoscale devices: Application to solar cells and molecular wires. Theor. Chem. Acc..

[16] De Angelis F., Fantacci S., Selloni A. (2008). Alignment of the dye’s molecular levels with the TiO(2) band edges in dye-sensitized solar cells: A DFT-TDDFT study. Nanotechnology.

[17] Seo K.D., Song H.M., Lee M.J., Pastore M., Anselmi C., de Angelis F., Nazeeruddin M.K., Gräetzel M., Kim H.K. (2011). Coumarin dyes containing low-band-gap chromophores for dye-sensitised solar cells. Dyes Pigment..

[18] Hara K., Sayama K., Arakawa H., Ohga Y., Shinpo A., Suga S. (2001). A coumarin-derivative dye sensitized nanocrystalline TiO_2_ solar cell having a high solar-energy conversion efficiency up to 5.6%. Chem. Commun..

[19] Hara K., Miyamoto K., Abe Y., Yanagida M. (2005). Electron transport in coumarin-dye-sensitized nanocrystalline TiO_2_ electrodes. J. Phys. Chem. B.

[20] Wang Z.S., Cui Y., Hara K., Dan-Oh Y., Kasada C., Shinpo A. (2007). A High-light-harvesting-efficiency coumarin dye for stable dye-sensitized solar cells. Adv. Mater..

[21] Wang Z.S., Cui Y., Dan-oh Y., Kasada C., Shinpo A., Hara K. (2007). Thiophene-functionalized coumarin dye for efficient dye-sensitized solar cells: Electron lifetime improved by coadsorption of deoxycholic acid. J. Phys. Chem. C.

[22] Ooyama Y., Harima Y. (2009). Molecular designs and syntheses of organic dyes for dye-sensitized solar cells. Eur. J. Org. Chem..

[23] Hara K., Sato T., Katoh R., Furube A., Ohga Y., Shinpo A., Suga S., Sayama K., Sugihara H., Arakawa H. (2003). Molecular design of coumarin dyes for efficient dye-sensitized solar cells. J. Phys. Chem. B.

[24] Kurashige Y., Nakajima T., Kurashige S., Hirao K. (2007). Theoretical investigation of the excited states of coumarin dyes for dye-sensitized solar cells. J. Phys. Chem. A.

[25] Preat J., Loos P.-F., Assfeld X., Jacquemin D., Perpète E.A. (2007). A TD-DFT investigation of UV spectra of pyranoïdic dyes: A NCM *vs*. PCM comparison. J. Mol. Struct. Theochem..

[26] Zhang X., Zhang J.-J., Xia Y.-Y. (2008). Molecular design of coumarin dyes with high efficiency in dye- sensitized solar cells. J. Photochem. Photobiol. A Chem..

[27] Sanchez-de-Armas R., San Miguel M.A., Oviedo J., Sanz J.F. (2012). Coumarin derivatives for dye sensitized solar cells: A TD-DFT study. Phys. Chem. Chem. Phys..

[28] Kondov I., Wang H., Thoss M. (2006). Computational study of titanium (IV) complexes with organic chromophores. Int. J. Quantum Chem..

[29] Hohenberg P., Kohn W. (1964). Inhomogeneous electron gas. Phys. Rev..

[30] Kohn W., Sham L.J. (1965). Self-consistent equations including exchange and correlation effects. Phys. Rev..

[31] Parr R.G., Yang W. (1989). Density-Functional Theory of Atoms and Molecules.

[32] Becke A.D. (1988). Density-functional exchange-energy approximation with correct asymptotic behavior. Phys. Rev. A.

[33] Lee C., Yang W., Parr R.G. (1988). Development of the colle-salvetti correlation-energy formula into a functional of the electron density. Phys. Rev..

[34] Hay P.J., Wadt W.R. (1985). *Ab initio* effective core potentials for molecular calculations. Potentials for K to Au including the outermost core orbitals. J. Chem. Phys..

[35] Becke A.D. (1993). Density-functional thermochemistry. III. The role of exact exchange. J. Chem. Phys..

[36] Godbout N., Salahub D.R., Andzelm J., Wimmer E. (1992). Optimization of Gaussian-type basis sets for local spin density functional calculations. Part I. Boron through neon, optimization technique and validation. Can. J. Chem..

[37] Casida M.E., Jamorski C., Casida K.C., Salahub D.R. (1998). Molecular excitation energies to high-lying bound states from time-dependent Density-Functional Response theory: Characterization and correlation of the time dependent local density approximation ionization threshold. J. Chem. Phys..

[38] Barone V., Cossi M. (1998). Quantum calculation of molecular energies and energy gradients in solution by a conductor solvent model. J. Phys. Chem. A.

[39] Tomasi J., Mennucci B., Cammi R. (2005). Quantum mechanical continuum solvation models. Chem. Rev..

[40] Frisch M.J., Trucks G.W., Schlegel H.B., Scuseria G.E., Robb M.A., Cheeseman J.R., Montgomery J.A., Vreven T., Kudin K.N., Burant J.C. Gaussian 03 Citation.

[41] Badaeva E., Albert V.V., Kilina S., Koposov A., Sykora M., Tretiak S. (2010). Effect of deprotonation on absorption and emission spectra of Ru(II)-bpy complexes functionalized with carboxyl groups. Phys. Chem. Chem. Phys..

[42] Fantacci S., de Angelis F., Selloni A. (2003). Absorption spectrum and solvatochromism of the [Ru(4,4′-COOH-2,2′-bpy)_2_(NCS)_2_] molecular dye by time dependent density functional theory. J. Am. Chem. Soc..

[43] Gueymard C. (2004). The sun’s total and spectral irradiance for solar energy applications and solar radiation models. Sol. Energy.

[44] Peter L.M. (2007). Characterization and modeling of dye-sensitized solar cells. J. Phys. Chem. C.

[45] Matthews D., Infelta P., Gratzel M. (1996). Calculation of the photocurrent-potential characteristic for regenerative, sensitized semiconductor electrodes. Sol. Energy Mater. Sol. Cells.

[46] Anderson N.A., Lian T. (2004). Ultrafast electron injection from metal polypyridyl complexes to metal-oxide nanocrystalline thin films. Coord. Chem. Rev..

[47] Koops S.E., O’Regan B.C., Barnes P.R.F., Durrant J.R. (2009). Parameters influencing the efficiency of electron injection in dye-sensitized solar cells. J. Am. Chem. Soc..

[48] Chen R., Yang X., Tian H., Wang X., Hagfeldt A., Sun L. (2007). Effect of tetrahydroquinoline dyes structure on the performance of organic dye-sensitized solar cells. Chem. Mater..

[49] Oprea C.I., Dumbrava A., Enache I., Lungu J., Georgescu A., Moscalu F., Oprea C., Gîrţu M.A. (2011). Role of energy level alignment in solar cells sensitized with a metalfree organic dye: A combined experimental and theoretical approach. Phys. Status Solidi.

[50] Oprea C.I., Panait P., Lungu J., Stamate D., Dumbrava A., Cimpoesu F., Gîrţu M.A. (2013). DFT study of binding and electron transfer from a metal-free dye with carboxyl, hydroxyl and sulfonic anchors to a titanium dioxide nanocluster. Int. J. Photoenergy.

[51] Lazzeri M., Vittadini A., Selloni A. (2001). Structure and energetics of stoichiometric TiO_2_ anatase surfaces. Phys. Rev. B.

[52] Selloni A. (2008). Crystal growth—Anatase shows its reactive side. Nat. Mater..

[53] Horn M., Schwerdtfeger C.F., Meagher E.P. (1972). Refinement of the structure of anatase at several temperatures. Z. Krist..

[54] Lazzeri M., Selloni A. (2001). Stress-driven reconstruction of an oxide surface: The anatase TiO_2_(001)-(1 × 4) surface. Phys. Rev. Lett..

[55] De Angelis F., Fantacci S., Selloni A., Nazeeruddin M.K., Gratzel M. (2010). First-principles modeling of the adsorption geometry and electronic structure of Ru(II) dyes on extended TiO_2_ substrates for dye-sensitized solar cell applications. J. Phys. Chem. C.

[56] Pastore M., de Angelis F. (2012). Computational modelling of TiO_2_ surfaces sensitized by organic dyes with different anchoring groups: Adsorption modes, structure and implication for electron injection/recombination. Phys. Chem. Chem. Phys..

[57] Vittadini A., Selloni A., Rotzinger F.P., Gratzel M. (2000). formic acid adsorption on dry and hydrated TiO_2_ anatase (101) surfaces by DFT calculations. J. Phys. Chem. B.

[58] Srinivas K., Yesudas K., Bhanuprakash K., Rao V.J., Giribabu L. (2009). A combined experimental and computational investigation of anthracene based sensitizers for DSSC: Comparison of cyanoacrylic and malonic acid electron withdrawing groups binding onto the TiO_2_ anatase (101) surface. J. Phys. Chem. C.

[59] Leon C.P., Kador L., Peng B., Thelakkat M. (2006). Characterization of the adsorption of Ru-bpy dyes on mesoporous TiO_2_ films with UV-Vis, Raman, and FTIR spectroscopies. J. Phys. Chem. B.

[60] Martsinovich N., Troisi A. (2011). High-throughput computational screening of chromophores for dye-sensitized solar cells. J. Phys. Chem. C.

[61] Lungu J., Oprea C.I., Dumbrava A., Enache I., Georgescu A., Rădulescu C., Ioniţă I., Cimpoca G.V., Gîrţu M.A. (2010). Heterocyclic azodyes as pigments for dye sensitized solar cells—A combined experimental and theoretical study. J. Optoelectr. Adv. Mater..

