# Supplementary material for: Density Functional Theory (DFT) Study of Coumarin-based Dyes Adsorbed on TiO2 Nanoclusters—Applications to Dye-Sensitized Solar Cells
Source: Materials (Basel). 2013 Jun 10;6(6):2372–92. doi: 10.3390/ma6062372 (PMC5458949; doi:10.3390/ma6062372)
Supplement: Supplementary File 1 [file materials-06-02372-s001.pdf]

Materials **2013**, *6*, 2372-2392; doi:10.3390/ma6062372

OPEN ACCESS

materials

ISSN 1996-1944

www.mdpi.com/journal/materials

Article

Supplementary Information

Table S1. Wavelength and oscillator strength of the main transitions in the spectrum for the three coumarin-based dyes, in neutral and deprotonated anionic form, calculated by TD-DFT (at B3LYP/DZVP level) in various solvents.

Dye	Dye form	Solvent	Wavelength (nm)	Oscillator strength
C343	Deprotonated	water	413.57	0.5373
		methanol	405.24	0.5564
		ethanol	404.89	0.5658
	Neutral	water	423.67	0.7468
		methanol	419.77	0.7352
		ethanol	420.56	0.7457
NKX-2398	Deprotonated	water	438.11	0.7819
		methanol	435.00	0.8136
		ethanol	435.63	0.8195
	Neutral	water	454.22	1.0980
		methanol	449.83	1.0963
		ethanol	450.39	1.1072
NKX-2311	Deprotonated	water	490.55	1.4381
		methanol	488.06	1.4755
		ethanol	488.53	1.4894
	Neutral	water	534.67	1.5050
		methanol	530.48	1.5270
		ethanol	531.72	1.5417

Figure S1. Simulated UV-Vis absorption spectra of neutral and deprotonated C343 dyes, calculated by TD-DFT, in various solvents. The spectral lines were convoluted with Gaussian distributions of 20 nm linewidth at half maximum.

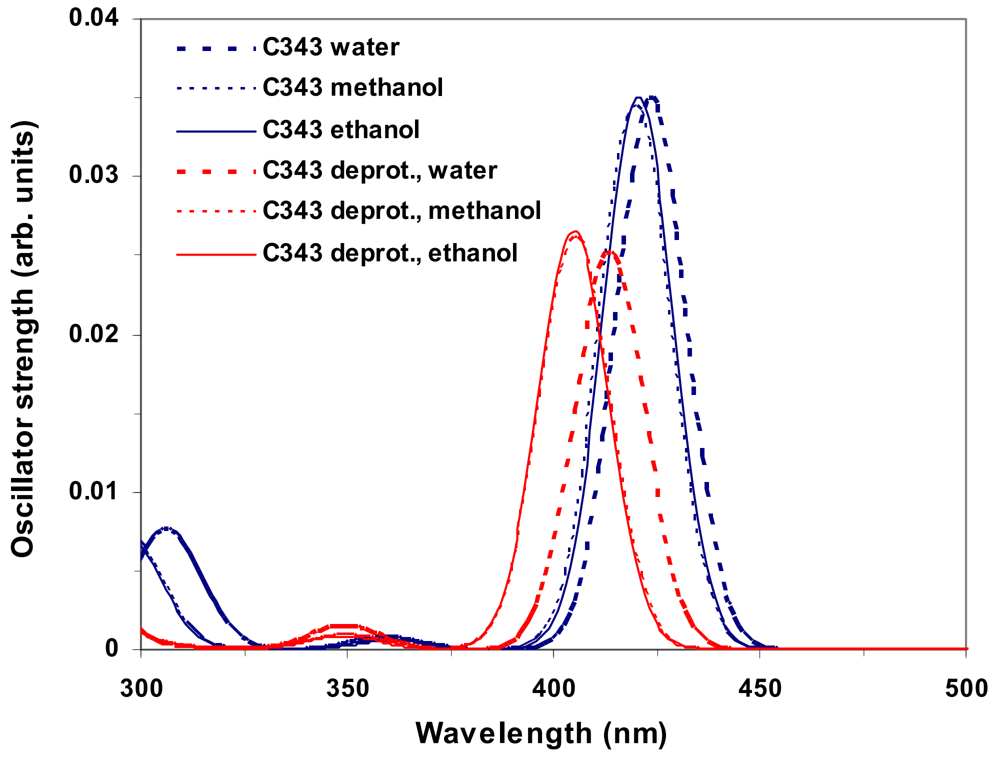

Figure S2. Simulated UV-Vis absorption spectra of neutral and deprotonated NKX-2398 dyes, calculated by TD-DFT, in various solvents. The spectral lines were convoluted with Gaussian distributions of 20 nm linewidth at half maximum.

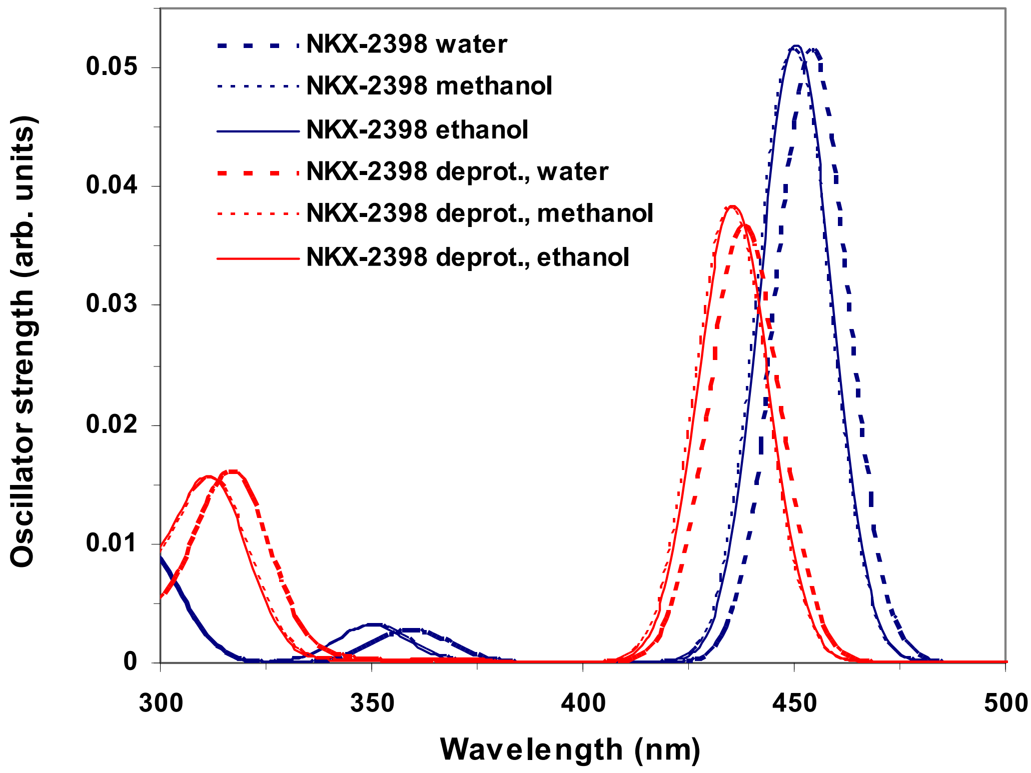

Figure S3. Simulated UV-Vis absorption spectra of neutral and deprotonated NKX-2311 dyes, calculated by TD-DFT, in various solvents. The spectral lines were convoluted with Gaussian distributions of 20 nm linewidth at half maximum.

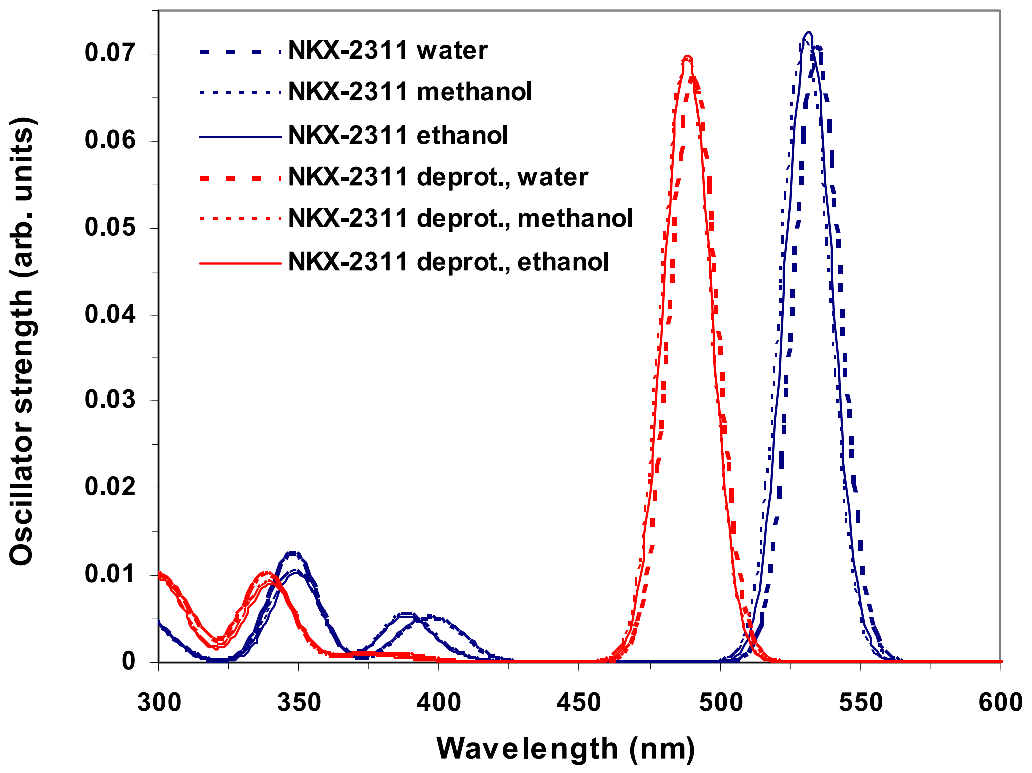

© 2013 by the authors; licensee MDPI, Basel, Switzerland. This article is an open access article distributed under the terms and conditions of the Creative Commons Attribution license (http://creativecommons.org/licenses/by/3.0/).
